# Periodontal disease increases the severity of chronic obstructive pulmonary disease: a Mendelian randomization study

**DOI:** 10.1186/s12890-024-03025-6

**Published:** 2024-05-03

**Authors:** Bao-Ling Zhao, Fei-Yan Yu, Zhen-Ni Zhao, Rong Zhao, Qian-Qian Wang, Jia-Qi Yang, Yu-Kai Hao, Zi-Qian Zhang, Xue-Jun Ge

**Affiliations:** 1 Shanxi Medical University School and Hospital of Stomatology, No.63 New South Road Yingze District Taiyuan, Taiyuan, 030001 People’s Republic of China; 2 Shanxi Province Key Laboratory of Oral Diseases Prevention and New Materials, Taiyuan, China; 3 Department of Rheumatology, The Second Hospital of Shanxi Medical University, Taiyuan, Shanxi China

**Keywords:** Mendelian randomization analysis, Periodontitis, Pulmonary disease, Chronic obstructive, Risk factors, Polymorphism, Single nucleotide

## Abstract

**Background:**

Recent research suggests that periodontitis can increase the risk of chronic obstructive pulmonary disease (COPD). In this study, we performed two-sample Mendelian randomization (MR) and investigated the causal effect of periodontitis (PD) on the genetic prediction of COPD. The study aimed to estimate how exposures affected outcomes.

**Methods:**

Published data from the Gene-Lifestyle Interaction in the Dental Endpoints (GLIDE) Consortium’s genome-wide association studies (GWAS) for periodontitis (17,353 cases and 28,210 controls) and COPD (16,488 cases and 169,688 controls) from European ancestry were utilized. This study employed a two-sample MR analysis approach and applied several complementary methods, including weighted median, inverse variance weighted (IVW), and MR-Egger regression. Multivariable Mendelian randomization (MVMR) analysis was further conducted to mitigate the influence of smoking on COPD.

**Results:**

We chose five single-nucleotide polymorphisms (SNPs) as instrumental variables for periodontitis. A strong genetically predicted causal link between periodontitis and COPD, that is, periodontitis as an independent risk factor for COPD was detected. PD (OR = 1.102951, 95% CI: 1.005–1.211, *p* = 0.039) MR-Egger regression and weighted median analysis results were coincident with those of the IVW method. According to the sensitivity analysis, horizontal pleiotropy’s effect on causal estimations seemed unlikely. However, reverse MR analysis revealed no significant genetic causal association between COPD and periodontitis. IVW (OR = 1.048 > 1, 95%CI: 0.973–1.128, *p* = 0.2082) MR Egger (OR = 0.826, 95%CI:0.658–1.037, *p* = 0.1104) and weighted median (OR = 1.043, 95%CI: 0.941–1.156, *p* = 0.4239). The results of multivariable Mendelian randomization (MVMR) analysis, after adjusting for the confounding effect of smoking, suggest a potential causal relationship between periodontitis and COPD (*P* = 0.035).

**Conclusion:**

In this study, periodontitis was found to be independent of COPD and a significant risk factor, providing new insights into periodontitis-mediated mechanisms underlying COPD development.

**Supplementary Information:**

The online version contains supplementary material available at 10.1186/s12890-024-03025-6.

## Introduction

Periodontitis (PD) is a frequent chronic inflammatory disorder with clinical manifestations of radiologically assessed alveolar bone loss, accompanying clinical attachment loss, gingival bleeding, and periodontal pocket formation [1]. Host immunophysiological disruption and genetic factors play an important role in the development of PD [2, 3]. Recently, genetic susceptibility factors were found to play a role in the development of PD. For instance, Matthias Munz et al. have identified a correlation between genetic variants in the loci SIGLEC5 and DEFA1A3 and the risk of PD [4].

Chronic obstructive pulmonary disease (COPD) is a chronic inflammatory disease characterized by restriction of expiratory airflow and persistent lung parenchymal obstruction, along with emphysematous lung destruction [5]. Changes in lung microecology, autoimmune components of the disease, and environmental risk factors together contribute to the development of COPD. In addition to the above, genetic susceptibility has been implicated in COPD [6].

Although COPD primarily affects the lungs, it is now considered as a multi-component disease, often co-existing with other disorders such as PD [7]. Similarities between the two diseases exist, with neutrophil infiltration, altered protease/anti-protease, and redox state balance playing a key role [8]. Several observational studies show an independent and significant link between PD and COPD. For example, some retrospective studies show that the development of PD increases the risk of death among COPD patients [9, 10]. Recently, a systematic review including two cross-sectional studies and a case-control study found that frequent COPD exacerbations were strongly associated with PD [11]. However, in contrast, Arianne K Baldomero and Zhou X, concluded that periodontal health status was not associated with a worsened COPD state [12, 13]. However, observational studies using cross-sectional or case-control methods suffer from the disadvantages of bias, confounding factors, and reverse causality. Therefore, clarifying the genetic causal relationship between PD and COPD risk is important for assessing the pathogenesis of COPD.

Mendelian randomization (MR) is a causal analysis method that exploits natural randomization in the generation of individual genetic constituents. MR uses genetic data including single nucleotide polymorphisms (SNPs) that are related to exposure, as instrumental variables to assess the causality of the association between exposure or risk factors and the outcome of interest. MR analysis can overcome the above-mentioned limitations of observational studies in causality investigations and can compensate for the disadvantages of randomized controlled trials (RCTs) including high human and material consumption and ethical challenges. A new perspective unlike traditional observational studies and RCTs is presented [14]. We conducted a two-sample MR study with data from genome-wide association studies (GWAS) to investigate if there is a genetically predicted causal relationship between PD and COPD, thus facilitating the prevention and treatment of these patients.

## Methods

### Study design

MR is a causal analysis method based on Mendel’s segregation and independent assortment laws [15]. This study uses a two-sample MR design to estimate the causal influence of exposure on the outcome using GWAS summary data from two independent studies. Three assumptions underpin the MR design. First, genetic variations are consistently linked to exposure. Second, no confounders are related to genetic variations. Third, apart from the exposure, genetic variations do not affect the outcomes. MR is a useful technique in genetic epidemiology because it provides a trustworthy approach to investigating causal inference.

**Fig. 1:**
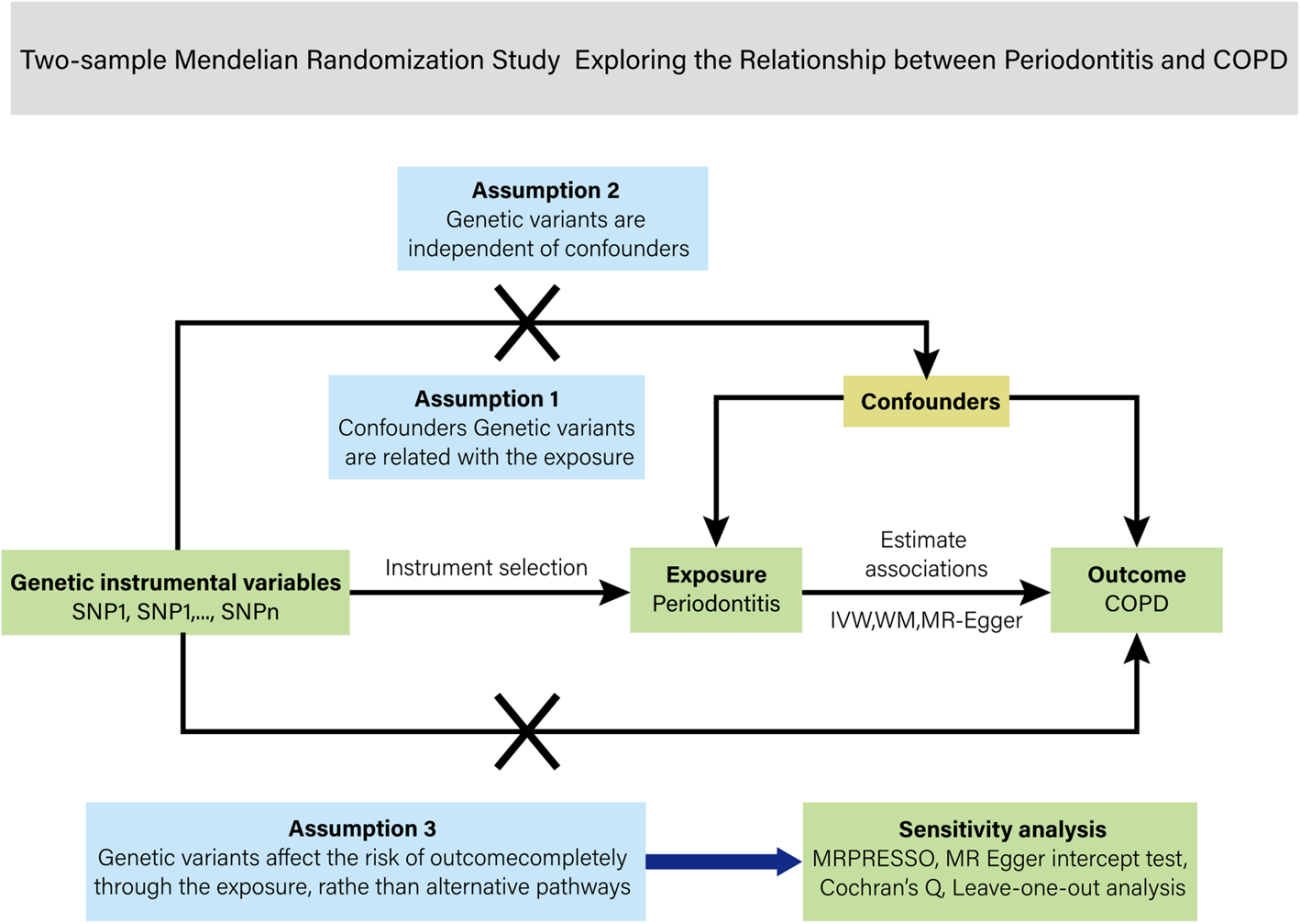
The schematic depiction of two-sample Mendelian randomization design. Valid genetic instrumental variations that satisfy the three assumptions required for Mendelian randomization

Our study design, with its 3 above-mentioned key components, is shown in Fig. 1.

### Data sources

Data on the genetic association of SNPs with PD were obtained from the latest and largest GWAS meta-analysis of the Gene-Lifestyle Interaction in the Dental Endpoints (GLIDE) Consortium, with a total sample size of 17,353 clinical cases and 28,210 controls (PMID:31235808) [16]. Patients with PD were as defined based on the Centers for Disease and Control and Prevention/American Academy of Periodontology (CDC/AAP) [17] or Community Periodontal Index (CPI) or by diagnostic classification of PD reported by study participants [16]. COPD statistics were obtained from the publicly available GWAS dataset from European ancestry, including 16,488 cases and 169,688 controls (PMID:33106845) [18]. We conducted a two-sample MR study using PD and COPD as the exposure and outcome, respectively.

### Selection of the instrumental variables

To satisfy the first assumption, we screened SNPs based on a *P*-value of 5 × 10^−6^ and used them as instrumental variables to identify independent SNPs associated substantially with PD. Second, we utilized the clumping approach with *R*^*2*^ < 0.001 and a window size of 10,000 kb to eliminate SNPs showing a strong linkage disequilibrium (LD). SNPs with effect allele frequency (EAF) of more than 0.01 were eliminated as well. The first condition was further verified by calculating the *F-*statistic and the explained phenotypic variance. IVs with an *F*-statistic of less than 10 were deemed weak instruments and eliminated from MR analysis [19]. To ensure that the second assumption was met, we used the PhenoScanner database for formerly reported correlations between instrumental SNPs (and LD proxies) and a potential confounding factor. These confounders included obesity and type 2 diabetes, BMI, alcohol, smoking, and rheumatoid arthritis. We used (*P* < 1e-5, r2 > 0.8) selection thresholds to strengthen instrument analyses [20]. Considering the third assumption to ensure that genetic variants did not influence the outcome through any variable other than exposure, the instrumental variables screened in PD were also selected in COPD. Finally, we reconciled the exposure and result datasets. Furthermore, a lack of instrument-exposure relationship may have diminished the validity of the MR relevance assumption.

### Statistical analysis

To analyze the potential causal relationship between exposure and outcome, we used the R package, TwoSampleMR (0.5.6) [21]. In this study, to quantify the causal effects of exposure on its outcome, we used several complementary approaches, including the inverse variance weighted (IVW), MR-Egger regression [22], and weighted median [23]. IVW approach used to pool Wald ratios was the primary method of analysis [24]. IVW is a weighted regression of the combined SNP-exposure and SNP-outcome associations. If all of the IVs are valid or horizontal pleiotropy is balanced, its outcome is robust. Finally, given the strong correlation between smoking and COPD, we also conducted multivariable Mendelian randomization (MVMR) analysis to eliminate the interference of smoking-related phenotypes.

We used the Cochran’s Q-statistic from the IVW and MR Egger methods to test for heterogeneity (variability in causal estimates derived for each SNP) between the causal estimates of individual SNPs [25]. MR-Egger regression was used to assess the likelihood of horizontal pleiotropy. The intercept in the MR-Egger test represented the average direct impact of a variant on the results, while the slope represented the potential causal effect. To find horizontal pleiotropic outliers and identify whether there were considerable differences in the causal effects before and after outlier filtering, MR-PRESSO (pleiotropy residual sum and outlier) was employed [26]. To determine whether a specific SNP instrument was driving the discrepancy in computed residual sum of squares (RSS) versus simulated expectations, the “leave-one-out” technique was utilized.

## Results

### Instrumental variable selection for PD and COPD

Genetic variation is used as a proxy measure for risk factors in the MR approach to estimate the causal impact of the concerned risk factor. Fifty-nine independent SNPs associated with PD were identified in the latest GWAS dataset (*p* < 5 × 10^−6^). After the removal of LD (R2 < 0.001 and window size, 10,000 kb) and effect alleles (EAF > 0.01), eight SNPs remained. The details of these eight SNPs with respect to PD are shown in Supplementary Table S1. Considering the common confounders of PD, we generated associations of SNPs based on the PhenoScanner search, excluding a confounder BMI-related SNP (rs2921075). Subsequently, SNPs that were absent and/or significant in GWAS for COPD were excluded (rs138868497); six of these SNPs were included in the analysis. Moreover, due to the difficulties in identifying the effect alleles for the exposure and outcome, palindromic SNPs (i.e., an allele of an SNP consisting of a base and its complementary base) were removed (rs4811024). Ultimately, valid instrumental variables that met the three MR conditions stated above were selected. Five SNPs were included in the MR analysis, namely rs10143801, rs151226594, rs73155039, rs76734229, and rs9954920 (Table 1). Finally, we reversed the exposure and outcome, considering COPD as the exposure and periodontitis as the outcome. Using the same selection criteria, we identified 32 SNPs for analyzing the reverse causal relationship between COPD and periodontitis.

**Table 1:** SNPs used as instruments variables and their association with the exposure and the outcome

SNP	EA/OA	EAF	PD			r2	***F***	COPD		
			BETA	SE	***P***-value			SE	***P***-value	BETA
periodontitis								COPD		
rs10143801	G/A	0.3258	0.084	0.0171	8.66 × 10^−7^	0.0031	141.667	0.0137	0.816	0.0032
rs151226594	G/T	0.0184	0.3671	0.0768	1.75 × 10^−6^	0.0049	222.876	0.0473	0.216	0.0585
rs73155039	G/A	0.0106	−0.8316	0.1757	2.22 × 10^−6^	0.0145	670.619	0.0538	0.065	−0.0992
rs76734229	A/G	0.0737	−0.1761	0.037	1.94 × 10^−6^	0.0042	193.733	0.0212	0.378	−0.0187
rs9954920	T/C	0.3572	0.0769	0.0163	2.37 × 10^−6^	0.0027	124.064	0.0125	0.581	−0.0069

*PD* periodontitis, *COPD* Chronic Obstructive Pulmonary Disease, *EA/OA* effect allele/reference allele, *EAF* effect allele frequency, *SE* standard error, *SNP* single nucleotide polymorphism. R2 = 2 × MAF × (1-MAF) × Beta; *F*-statistic = R2 (N-2)/(1-R2)

All *F*-statistic values were > 10, indicating no weak instrument bias.

### Sensitivity analysis supporting the causal association between PD and COPD

We employed the MR-Egger approach for measuring heterogeneity among the causal assessments of individual SNPs. Cochran’s Q-statistic showed no substantial indication of heterogeneity between SNPs (*P* = 0.9204 > 0.05). Among the IVW estimates, no substantial heterogeneity variability between Wald ratios was found. Analysis of the MR-Egger intercept revealed no directional pleiotropy (Table 2). The funnel plot also supported the above findings (Fig. S1).

**Table 2:** The heterogeneity and horizontal pleiotropy of individual SNPs

		Heterogeneity						MR-Egger test for horizontal pleiotropy		
		MR-Egger			IVW					
**Exposure**	**Outcome**	***Q***	***df***	***P***	***Q***	***df***	***P***	**Intercept**	**SE**	***P***
PD	COPD	0.493	3	0.920	1.805	4	0.772	−0.0132	0.0114	0.3351

*PD* periodontitis, *COPD* Chronic Obstructive Pulmonary Disease, *IVW* inverse-variance weighted, *MR* Mendelian randomization, *Q* heterogeneity statistic Q, *df* degree of freedom

**Fig. 2:**
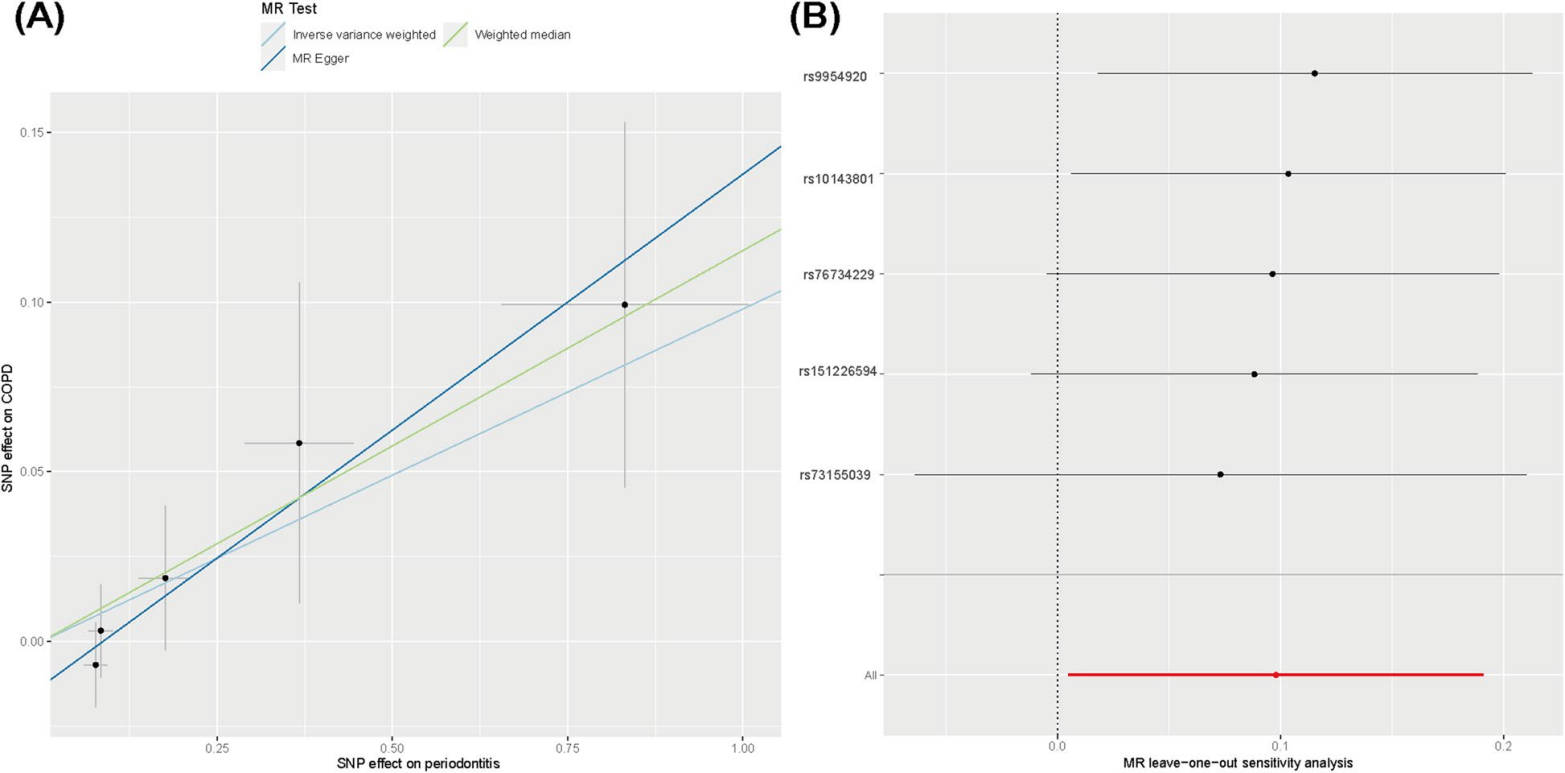
MR analysis and leave-one-out analysis of the causal effect of PD on COPD susceptibility (**A**) A scatter plot depicting the causal relationships between PD and COPD using various MR methods. Each line’s slope corresponds to the expected MR effect for each method. **B** The causal links between periodontitis and COPD are depicted using a leave-one-out plot. The leave-one-out figure depicted how the removal of a single variant altered the causal estimations (point with horizontal line) for the effect of periodontitis on COPD

Furthermore, the leave-one-out analysis showed no significant differences in PD and COPD causal estimations, indicating that none of the discovered causative links were driven by a single IV. The causal links between PD and COPD are depicted in the leave-one-out graphic (Fig. 2).

### The causal associations of PD with COPD

**Fig. 3:**
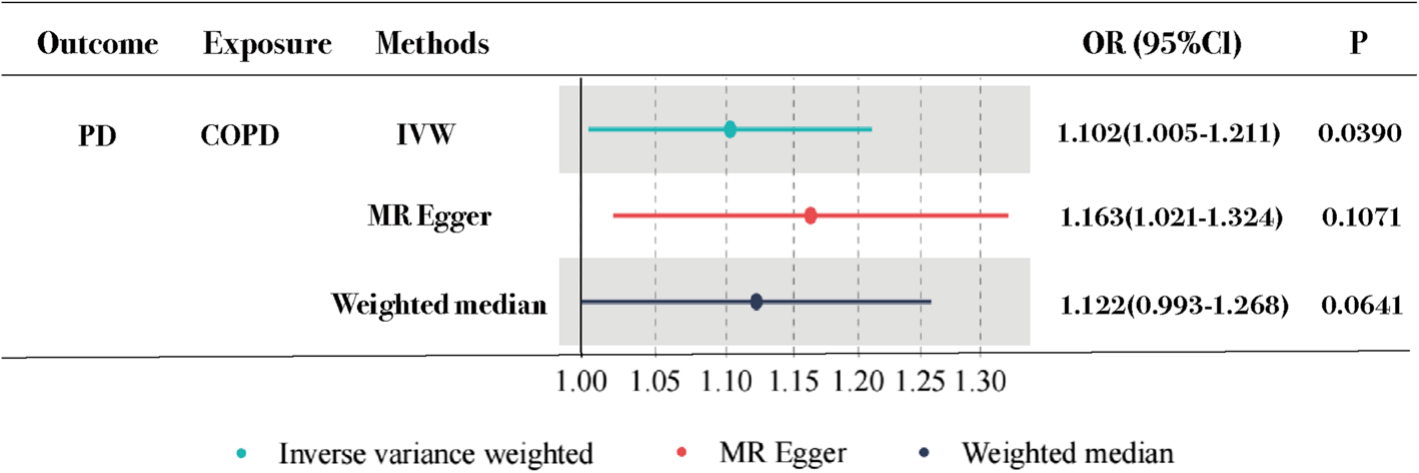
Comparison of Mendelian randomization results obtained using various approaches. PD: periodontitis; COPD: chronic obstructive pulmonary disease; IVW: inverse variance weighted; OR: odds ratio; CL: confidence interval

We used IVW, weighted median, and MR-Egger approaches for MR analysis. The results of MR analysis favored a significant causal association of PD with COPD. The IVW (OR = 1.102951 > 1, 95%CI: 1.005–1.211, *p* = 0.039) method showed statistical significance for PD as a notable risk factor for COPD. MR Egger (OR = 1.163, 95%CI:1.021–1.324, *p* = 0.1071) and weighted median (OR = 1.122, 95%CI: 0.993–1.268, *p* = 0.0641) methods showed results consistent with those of IVW. Estimated causal effects between PD and COPD using different MR methods are presented in Table 3 and Figs. 2 and 3.

**Table 3:** Three Mendelian randomization methods for the analysis of the causal relationship between periodontitis and COPD

Exposure	Outcome	Methods	***P***.value	OR	95%CI	
PD	COPD	IVW	*.0390*	*1.102*	1.005	1.211
		MR- Egger	*.1071*	*1.163*	1.021	1.324
		WM	*.0641*	*1.122*	0.993	1.268

*PD* periodontitis, *COPD* Chronic Obstructive Pulmonary Disease, *OR* odds ratio, *CI* confidence interval, *IVW* inverse-variance weighted, *MR* Mendelian randomization, *WM* Weighten median

### The causal associations of COPD with PD

We reversed the exposure and outcome, considering COPD as the exposure and periodontitis as the outcome. Using the same selection criteria, we identified 32 SNPs for analyzing the reverse causal relationship between COPD and periodontitis. The MR results indicate no significant genetically predicted association between COPD and periodontitis. IVW (OR = 1.048 > 1, 95%CI: 0.973–1.128, *p* = 0.2082) MR Egger (OR = 0.826, 95%CI:0.658–1.037, *p* = 0.1104) and weighted median (OR = 1.043, 95%CI: 0.941–1.156, *p* = 0.4239)(Table 4) According to Cochran’s Q-statistic, there was no notable evidence of heterogeneity among the SNPs. (MR-Egger *p* = 0.260 IVW *p* = 0.132) Additionally, the MR-Egger intercept test results effectively ruled out any directional pleiotropy. (*p* = 0.462) Furthermore, the leave-one-out sensitivity analysis demonstrated that no specific SNP significantly contributed to the association between COPD and PD.(Supp.Fig.S2-S4).

**Table 4:** Three Mendelian randomization methods for the analysis of the causal relationship between COPD and periodontitis

Exposure	Outcome	Methods	***P***.value	OR	95%CI	
COPD	PD	IVW	.2082	1.048	0.974	1.129
		MR- Egger	.1104	0.826	0.658	1.037
		WM	.4239	1.043	0.941	1.156

*PD* periodontitis, *COPD* Chronic Obstructive Pulmonary Disease, *OR* odds ratio, *CI* confidence interval, *IVW* inverse-variance weighted, *MR* Mendelian randomization, *WM* Weighten median

### MVMR analysis

Considering the potential contribution of smoking to the progression of COPD, we employed multivariable Mendelian randomization (MVMR) to examine significant associations, adjusting for the impact of smoking on COPD. The results of the multivariable MR analysis indicate that after adjusting for the confounding effects of smoking, a genetic causal relationship between periodontitis and COPD may be inferred. (*P* = 0.035<0.05)(Table 5).

**Table 5:** The MVMR results with adjusting the smoking exposure

Exposure	Outcome	nSNP	Beta	SE	OR(95%CI)	***P***-value
Smoking	COPD	47	0.282	0.175	1.326 (0.983–1.669)	0.106
PD	COPD	47	0.127	0.060	1.135 (1.018–1.253)	0.035

*PD* periodontitis, *COPD* Chronic Obstructive Pulmonary Disease, *SE* standard error, *SNP* single nucleotide polymorphism, *OR* odds ratio, *CI* confidence interval

## Discussion

This is the first report of a two-sample MR analysis for determining the genetic causal relationship between PD and COPD. The MR study provided convincing evidence that PD is genetically causally associated with COPD and is an independent risk factor for COPD development.

The first association between dental health and COPD in community-dwelling populations was discovered in a study of 23,808 people by the National Health and Nutrition Examination Survey I (NHANESI) [27]. Observational studies suggest that acute exacerbation of PD is a key risk factor for COPD progression and is associated with high mortality in COPD patients [28–30]. Frank A Scannapieco and coworkers found that the more severe the periodontal attachment loss, the greater the risk of COPD (odds ratio: 1.35; 95% confidence interval: 1.07–1.71) [31]. Hayes et al. also found that PD, as measured by alveolar bone loss assessed by periapical radiographs, was an independent risk factor for COPD [32]. Niamh Kelly and ZeSheng Wu et al. also suggested that poor periodontal health was associated with worsened COPD [11, 33]. Recently, Liu Shu qin et al. identified a potential genetic crosstalk between PD and COPD [34]. PD was further identified as an important and independent risk factor for COPD based on a meta-analysis of 14 observational studies [35]. Finally, A meta-analysis comprising 75 survey studies revealed a significant positive correlation between periodontitis and COPD [36]. These findings provide preliminary evidence that PD is an important factor that promotes COPD.

The results of this MR study strongly supported a genetically predicted causal relationship between PD and COPD and demonstrated that the former is an important risk factor for the latter. Therefore, the potential pathophysiological relationship between PD and COPD warrants further investigation.

We believe that the causal relationship between these two diseases may be closely related to the following reasons. First, oral flora is an important factor in the causal relationship between PD and COPD. Previous studies implicated oral bacteria in lung infections. Through various masticatory motions, dental plaque shed into saliva may change the respiratory epithelium, allowing pulmonary pathogen colonies to adhere strongly and grow [37]. Andreea C Didilescu et al. showed that dental plaque may serve as a reservoir for respiratory bacteria [38], crucial for the advancement of COPD. Studies have identified common pathogens between PD and COPD, including *Porphyromonas gingivalis*, *Tannerella forsythia*, Haemophilus, and *Treponema denticola* [39]. Among these, *Porphyromonas gingivalis* is closely linked to the development and progression of periodontal disease, being a gram-negative anaerobic bacterium commonly colonizing periodontal pockets [40]. Nan Feng et al. found that it could migrate to the lungs, alter the pulmonary microbiota, and exacerbate chronic obstructive pulmonary disease (COPD) [41]. Improved dental health may reasonably lower morbidity in COPD patients [42]. Second, both diseases are also associated with inflammatory mediators. Local inflammation in the periodontium results in the release of several proinflammatory cytokines into the bloodstream, including interleukins IL-6, IL-1α, and IL-1β, interferon IFN-γ, and tumor necrosis factor (TNF-α) [43]. These inflammatory factors may be connected to respiratory disease infections. In animal and cell studies, neutrophils have been linked to the onset and progression of COPD, by releasing inflammatory mediators like neutrophil elastase and matrix metalloproteinases (MMPs) [10]. According to a review by Hajishengallis, diseases characterized by aberrant neutrophil functions have a higher associated frequency of PD, including neutrophil deficiency and autoimmune neutropenia [38]. Epidemiological studies have found evidence to support this relationship. Finally, it is noteworthy that Kaixin Xiong et al. first discovered the crucial role of the γδ T-M2 immune mechanism in mediating periodontitis-promoted COPD [44]. Additionally, Shuqin Liu et al. identified EPB41L4A-AS1 as a potential cross-interacting gene between the two diseases. Its downregulation activates the nuclear factor kappa B (NF-κB) signaling pathway and enhances inflammatory responses, which also plays a pivotal role in the pathogenesis of periodontitis-promoted COPD [34].

Our MR analysis has numerous advantages. First, environmental factors and behavior do not affect genetic variation. As a result, MR analysis decreases residual confounders and other biases, effectively reducing reverse causality [14]. To evaluate the possible genetic causal link between PD and COPD, we used the most recent and largest GWAS dataset for PD and COPD from a European-descent population, therefore minimizing the influence of population stratification. Additionally, MR also has the advantages of being cost-effective and having few ethical concerns [45]. Lastly, we used a two-sample design to estimate the link between genetic variant exposure and genetic variant outcomes from two independent comparable populations, yielding greater statistical power [46].

### Limitations

Some limitations also warrant consideration. First, the SNPs used were all from individuals of European ancestry. Such studies for other ethnicities are warranted. Currently, MR studies often assess the lifelong effects of risk factors on outcomes, and it is difficult to reveal the causal effects across the stages of disease development. Finally, given the varied definitions of PD employed in different studies, GWAS for consistent SNPs in PD is difficult.

## Conclusion

In conclusion, this study supports a genetically predicted association between PD and COPD through MR Analysis and considers the former to be a risk factor for the latter. Although the cause underlying this relationship needs to be studied further, we have provided genetic evidence that PD is linked to COPD. In the future, this genetic tool is expected to be valuable for preventing and treating COPD. Maintaining good dental hygiene may contribute to reducing the risk of developing chronic obstructive pulmonary disease (COPD). However, further research is needed to confirm these observational findings and explore potential mechanisms.

### Supplementary Information

**Supplementary Material 1.**

**Supplementary Material 2.**

## Data Availability

The periodontitis summary statistic data are available at https://data.bris.ac.uk/data/dataset/. The COPD summary data are available at https://www.ebi.ac.uk/gwas/studies/GCST90016594

## Abbreviations

COPD: Chronic obstructive pulmonary disease
PD: Periodontitis
MR: Mendelian randomization
IVW: Inverse variance weighted
SNPs: Single-nucleotide polymorphisms
