# Supplementary material for: Periodontal disease increases the severity of chronic obstructive pulmonary disease: a Mendelian randomization study
Source: BMC Pulm Med. 2024 May 3;24:220. doi: 10.1186/s12890-024-03025-6 (PMC11071140; doi:10.1186/s12890-024-03025-6)
Supplement: Supplementary file 1 — Supplementary Material 1. [file 12890_2024_3025_MOESM1_ESM.docx]

Supplementary Figure


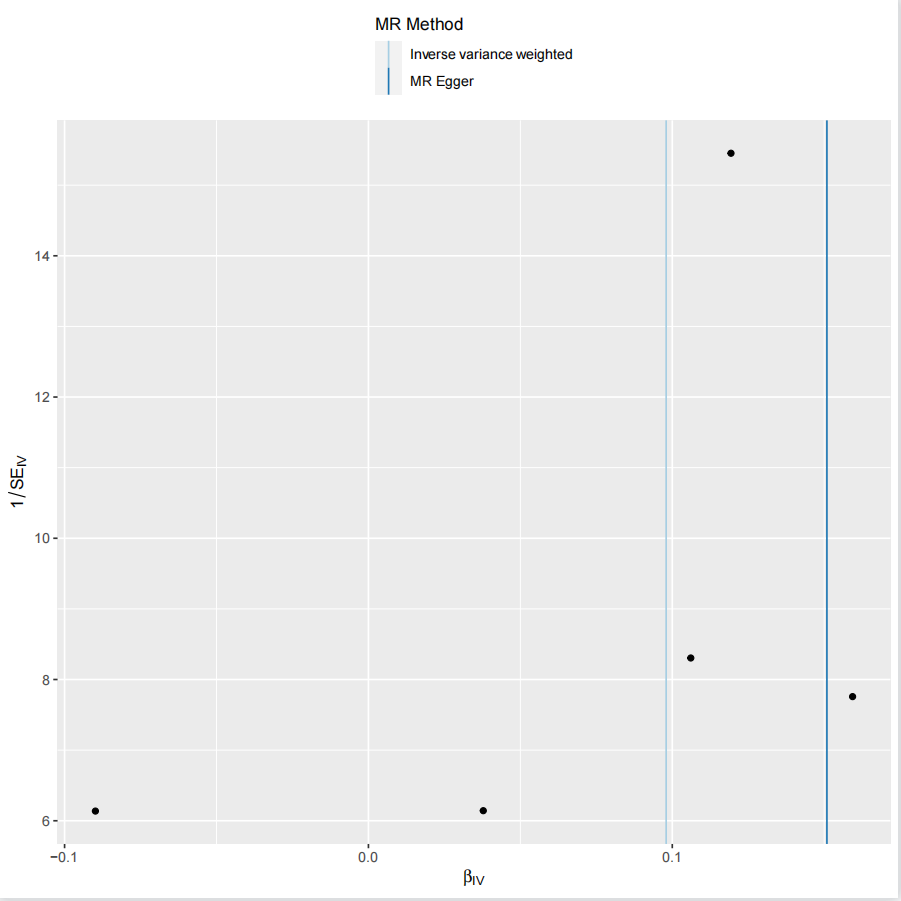


**Figure S1.** Funnel plot of the causal relationships between periodontitis and COPD. The funnel plot demonstrated the symmetry of causal estimates across all instrumental variables.


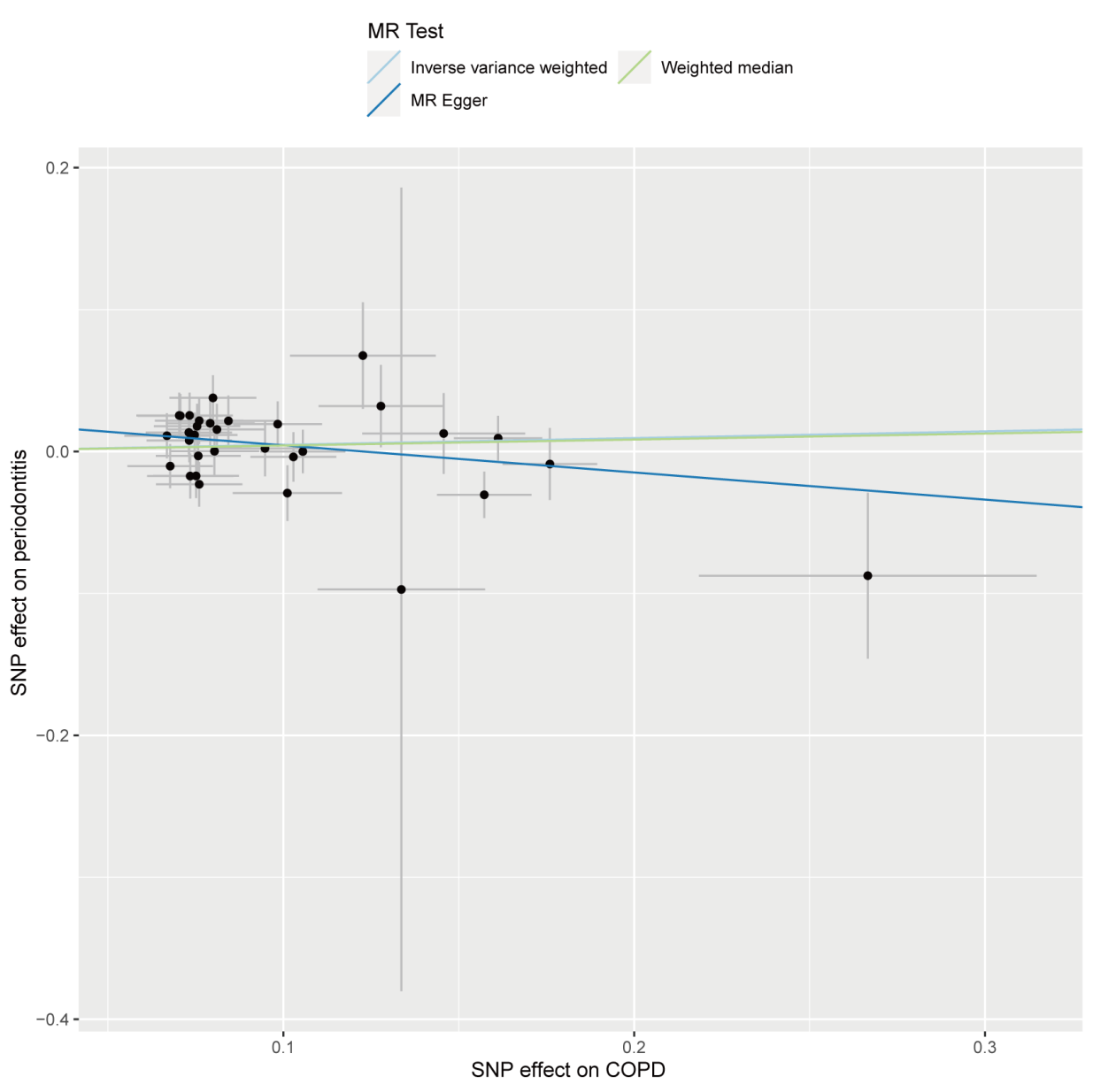


**Figure S2.**Depicting the causal estimates of COPD on periodontitis using different MR methods were utilized. Each point in the scatter plot represents a single SNP.


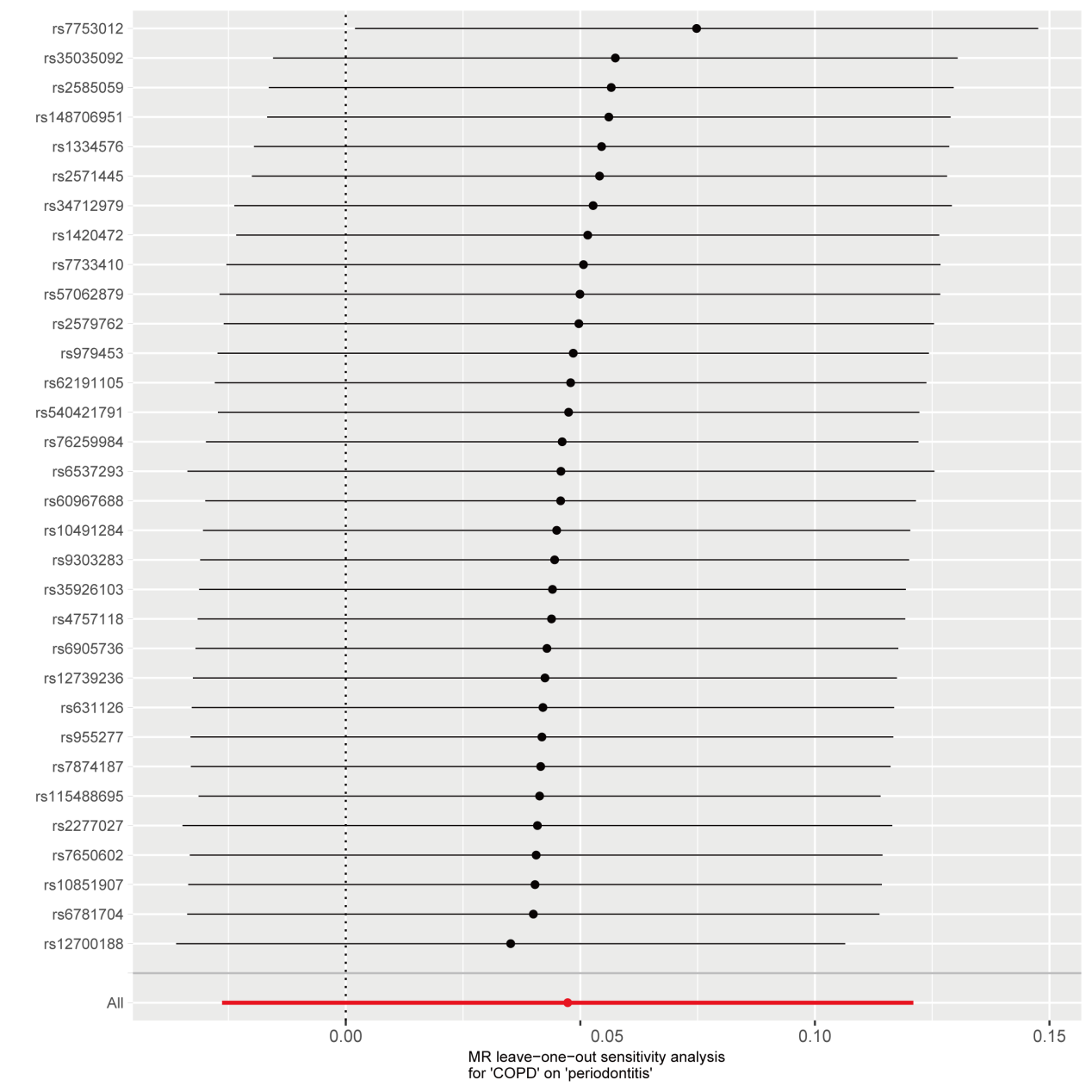


**Figure S3.** The causal links between COPD and periodontitis are depicted using a leave-one-out plot.


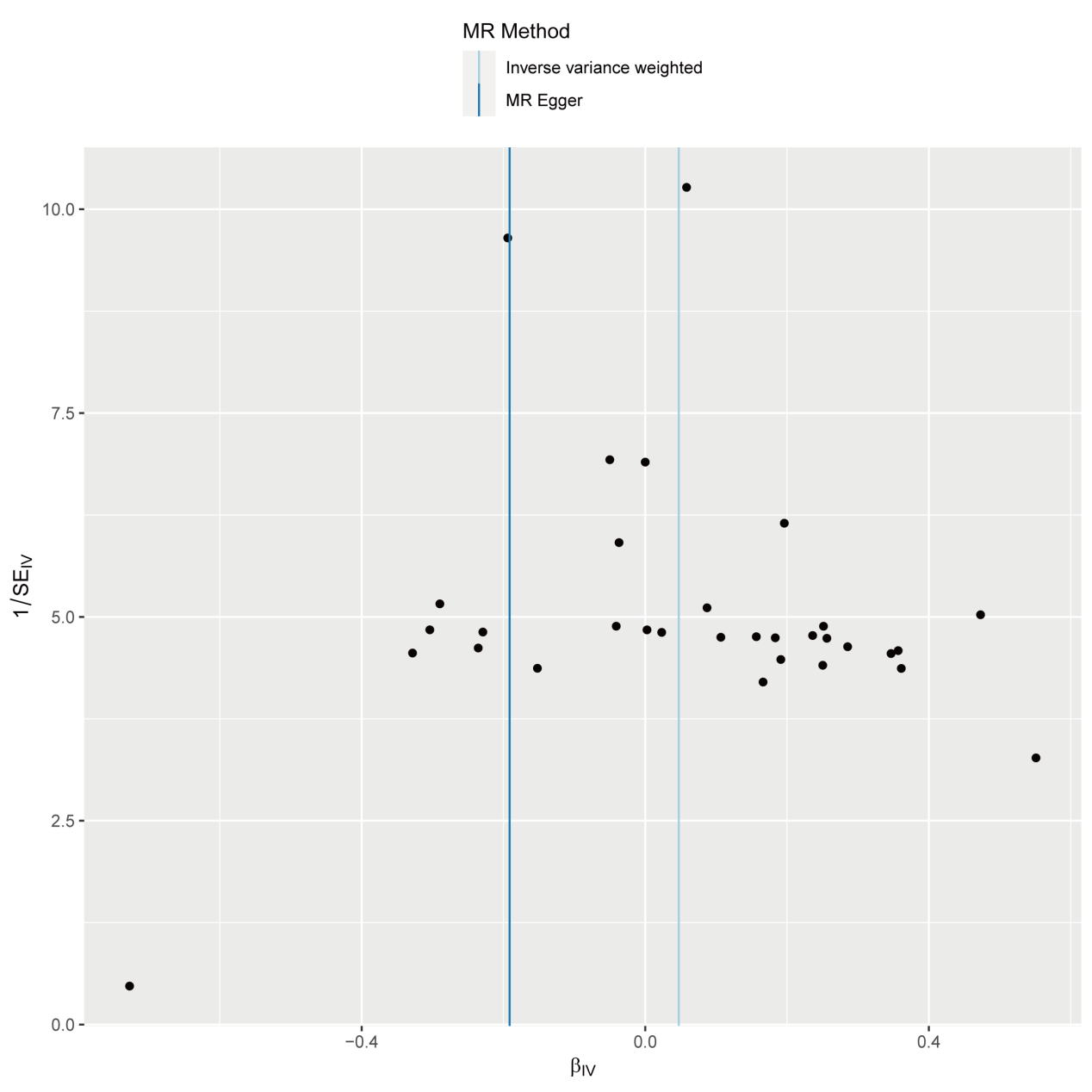


**Figure S4.** Funnel plot of the causal relationships between COPD and periodontitis.
