# Supplementary material for: Periodontal disease increases the severity of chronic obstructive pulmonary disease: a Mendelian randomization study
Source: BMC Pulm Med. 2024 May 3;24:220. doi: 10.1186/s12890-024-03025-6 (PMC11071140; doi:10.1186/s12890-024-03025-6)
Supplement: Supplementary file 2 — Supplementary Material 2. [file 12890_2024_3025_MOESM2_ESM.docx]

Supplementary Table

**Table S1** **Assessment of genetic instrumental variables for periodontitis**

| **SNP** | **OA** | **EA** | **BETA** | **SE** | ***P*-value** | **EAF** | **R^2^** | ***F*** |
| --- | --- | --- | --- | --- | --- | --- | --- | --- |
| rs151226594 | T | G | 0.3671 | 0.0768 | 1.75x10^-6^ | 0.0184 | 0.00487 | 222.876 |
| rs138868497 | T | C | -1.6387 | 0.3324 | 8.20x10^-7^ | 0.0103 | 0.05475 | 2638.854 |
| rs10143801 | A | G | 0.084 | 0.0171 | 8.66x10^-7^ | 0.3258 | 0.00310 | 141.667 |
| rs76734229 | G | A | -0.1761 | 0.037 | 1.94x10^-6^ | 0.0737 | 0.00423 | 193.733 |
| rs9954920 | C | T | 0.0769 | 0.0163 | 2.37x10^-6^ | 0.3572 | 0.00272 | 124.064 |
| rs4811024 | C | G | 0.1337 | 0.0292 | 4.62x10^-6^ | 0.8945 | 0.00337 | 154.236 |
| rs73155039 | A | G | -0.8316 | 0.1757 | 2.22x10^-6^ | 0.0106 | 0.01450 | 670.619 |
| rs2921075 | G | C | 0.0916 | 0.0183 | 5.44x10^-7^ | 0.6927 | 0.00357 | 163.334 |

SNP: single nucleotide polymorphism; EA/OA: effect allele/reference allele; EAF: effect allele frequency; SE: standard error; R2 = 2 × MAF × (1-MAF) × Beta; *F*-statistic = R2 (N-2)/(1-R2 )
